# Machine learning quantification of Amyloid-β deposits in the temporal lobe of 131 brain bank cases

**DOI:** 10.1186/s40478-024-01827-7

**Published:** 2024-08-17

**Authors:** Rebeca Scalco, Luca C. Oliveira, Zhengfeng Lai, Danielle J. Harvey, Lana Abujamil, Charles DeCarli, Lee-Way Jin, Chen-Nee Chuah, Brittany N. Dugger

**Affiliations:** 1 Department of Pathology and Laboratory Medicine, University of California Davis, 4645 2nd Ave. 3400a research building III, Sacramento, CA 95817 USA; 2 Department of Public Health Sciences, University of California Davis, School of Medicine, Sacramento, CA, USA; 3 Department of Neurology, University of California Davis, School of Medicine, Sacramento, CA, USA; 4 Department of Electrical and Computer Engineering, University of California Davis, Davis, CA, USA; 5 Present Address: Institute of Animal Pathology, Vetsuisse Faculty, University of Bern, Länggassstrasse 122, 3012 Bern, Switzerland

**Keywords:** Machine learning, Quantitative analysis, Neuropathology, Whole-slide imaging, Clinicopathological correlation

## Abstract

Accurate and scalable quantification of amyloid-β (Aβ) pathology is crucial for deeper disease phenotyping and furthering research in Alzheimer Disease (AD). This multidisciplinary study addresses the current limitations on neuropathology by leveraging a machine learning (ML) pipeline to perform a granular quantification of Aβ deposits and assess their distribution in the temporal lobe. Utilizing 131 whole-slide-images from consecutive autopsied cases at the University of California Davis Alzheimer Disease Research Center, our objectives were threefold: (1) Validate an automatic workflow for Aβ deposit quantification in white matter (WM) and gray matter (GM); (2) define the distributions of different Aβ deposit types in GM and WM, and (3) investigate correlates of Aβ deposits with dementia status and the presence of mixed pathology. Our methodology highlights the robustness and efficacy of the ML pipeline, demonstrating proficiency akin to experts’ evaluations. We provide comprehensive insights into the quantification and distribution of Aβ deposits in the temporal GM and WM revealing a progressive increase in tandem with the severity of established diagnostic criteria (NIA-AA). We also present correlations of Aβ load with clinical diagnosis as well as presence/absence of mixed pathology. This study introduces a reproducible workflow, showcasing the practical use of ML approaches in the field of neuropathology, and use of the output data for correlative analyses. Acknowledging limitations, such as potential biases in the ML model and current ML classifications, we propose avenues for future research to refine and expand the methodology. We hope to contribute to the broader landscape of neuropathology advancements, ML applications, and precision medicine, paving the way for deep phenotyping of AD brain cases and establishing a foundation for further advancements in neuropathological research.

**Supplementary Information:**

The online version contains supplementary material available at 10.1186/s40478-024-01827-7.

## Introduction

Postmortem histopathological evaluation of brain tissue plays a critical role in elucidating the progression of neurodegenerative diseases such as Alzheimer Disease (AD), providing invaluable information for both diagnosis and research [1, 2]. There have been numerous iterations over the years, with the most recent in 2012, of guidelines to denote and assess pathological features that serve as the gold standard for definitive diagnosis of AD and related dementia (ADRDs) [3–5]. Autopsy-based assessments, typically semi-quantitative, have provided significant insights into disease mechanisms, especially through clinicopathological correlations [6, 7].

Traditionally, neuropathology experts have relied on visual inspection of meticulously prepared brain tissue sections, typically subjected to immunohistochemical staining and mounted on glass slides [8]. These examinations are conventionally performed on brightfield microscopes, forming the cornerstone of neuropathological analysis and diagnosis. Advancements in imaging technology have recently revolutionized histopathological assessments. Whole-slide imaging (WSI) technology, enabled by slide scanners, has become a compelling modality for comprehensive pathological evaluation [9, 10]. By capturing ultra-high-resolution images, WSIs offer a computer-based interface paired with viewing/analysis software, allowing trained personnel to evaluate morphologies with the aid of computational tools [11, 12]. This integration of digitized slides and computer-based tools has introduced new approaches, including machine learning (ML) workflows to enhance efficiency and objectivity in the quantification of histopathological findings [2].

The extracellular aggregation of amyloid-β (Aβ) protein, in the form of Aβ plaques in the human brain is a cardinal pathological feature of AD [1, 13]. Diverse morphologies of Aβ deposits are distributed throughout the brain parenchyma [13], often coexisting with cerebral amyloid angiopathy (CAA), characterized by the accumulation of Aβ in the walls of blood vessels [14–16]. While Aβ deposits have predominantly been observed in the gray matter (GM), their presence in the white matter (WM) has been reported [17]. This intricate distribution pattern may provide valuable insights into the complex nature of AD pathology and further emphasizes the importance of investigating both GM and WM regions, to provide more precise measurements, in comprehensive neuropathological analyses.

Quantification of Aβ deposit distribution, particularly in the context of manual identification and segmentation of GM and WM, can be laborious and time-consuming [10]. Current criteria for evaluating Aβ-related pathologies’ distribution and density primarily rely on semi-quantitative scoring systems, which can have interrater variability [4, 16, 18–22]. This is compounded by the vast presence of Aβ deposits in brain tissue and the high gigapixel resolution of WSIs, posing challenges in simultaneously analyzing multiple WSIs due to computational power and storage limitations. Additionally, conventional ML approaches are hindered by the large image resolution, preventing the direct use of a single WSI as input to ML models for analysis [23]. To advance the understanding of AD progression and the relation of hallmark deposits to clinicopathologic features, an automated end-to-end system was developed to identify different Aβ morphologies, quantify their numbers, and visualize their distributions in both GM and WM [24]. Implementing such a harmonized workflow can enhance the efficiency and effectiveness of neuropathological evaluations, enabling persons to extract more comprehensive insights into AD’s evolving landscape and heterogeneity.

Convolutional neural networks (CNNs) have shown great promise in augmenting the identification of AD-related pathological features [25–27]. A recent study demonstrated the capability of a CNN to quantify Aβ deposits comparable to an expert [25] which has been validated in independent cohorts [28]. Furthermore, our group has demonstrated a type of CNN, modified ResNet-18 [29], can automatically and efficiently perform GM/WM segmentation [27]. In the present study, we evaluated over 100 cases from the University of California (UC), Davis Alzheimer Disease Research Center utilizing a CNN workflow to automate the quantification of Aβ deposits in the temporal lobe, both in GM and WM regions, as well as visualize the locations and distributions of each type of Aβ deposits.

## Methods

### Dataset selection / sample selection

**Fig. 1:**
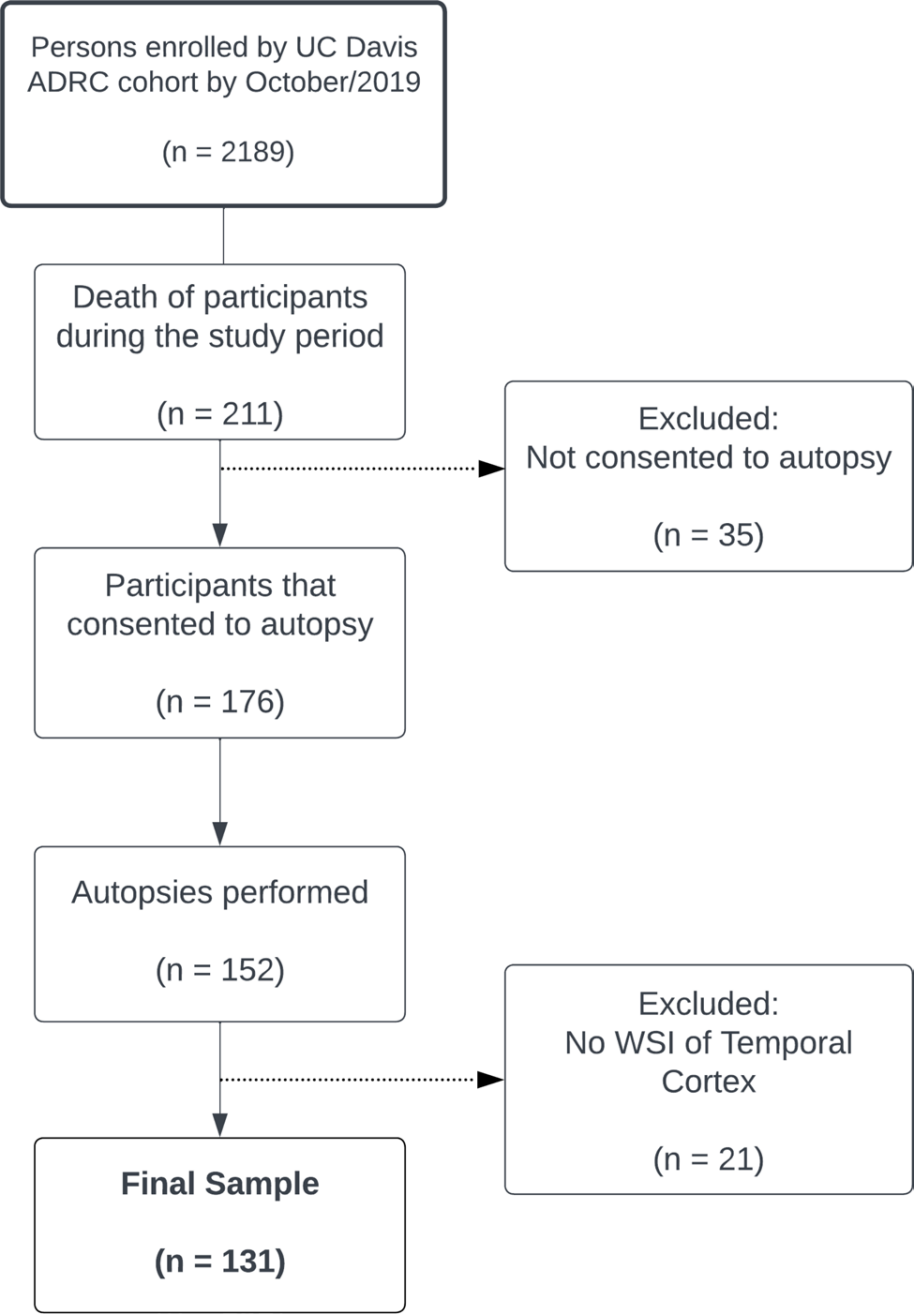
Flowchart of the study

Our cohort consisted of consecutive autopsied cases conducted between December 2012 and October 2019 at the University of California (UC), Davis (see Study Flowchart – Fig. 1). From these consecutive cases, available Aβ stained temporal lobe slides were scanned. This yielded a collection of 131 WSIs of the temporal lobe, lacking any personnel identifiers ensuring compliance with the Health Insurance Portability and Accountability Act (HIPAA). This study was approved by the Institutional Review Board (IRB) of the UC Davis, and written consent was obtained during life for autopsy for each participant. Details of this program have been previously published [30]. Information on participants’ sex, age, and self-reported race and/or ethnicity was obtained from forms provided by the National Alzheimer’s Coordinating Center (NACC) [31]. The inclusion criteria employed in this study were: (i) clinical/pathological criteria: inclusion of well-characterized clinical cases that exhibited a range of pathognomonic diagnostic histopathological features; (ii) technical criteria: inclusion of available samples with adequately stained tissue. The dataset encompasses a comprehensive range of cases, covering the entire spectrum of Alzheimer disease (AD) pathology burden, including individuals without cognitive impairment and cases with no to minimal of AD pathology. Cohort descriptions, including pathologic diagnoses, etc., are located in Table 1.

**Table 1:** Select demographics, select clinical comorbidities, and APOE e4 genotype status distribution of the study participants, divided by Alzheimer’s Disease Neuropathologic Change (ADNC) (*n* = 131)

	No/Not ADNC (*n* = 16)	Low ADNC (*n* = 30)	Intermediate ADNC (*n* = 23)	High ADNC (*n* = 62)	*P* value
**Demographic/Clinical data**					
**Age at death (years), mean (SD)**	85.9 (10.2)	86.9 (8.0)	86.7 (6.5)	84.0 (7.9)	0.31£
**Education attainment (years), mean (SD)**	14.3 (4.0)	12.9	15.5 (3.6)	14.8 (3.5)	0.063£
**Gender, (% female), N (%)**	7 (43.8%)	16 (53.3%)	12 (52.2%)	29 (46.7%)	0.89†
**Race / Ethnicity, N (%)**					
African American	2 (12.5%)	9 (30%)	0 (0%)	7 (11.3%)	**0.034§**
Asian	0 (0%)	0 (0%)	3 (13%)	3 (4.8%)	
Hispanic	2 (12.5%)	4 (13.3%)	2 (8.7%)	4 (6.5%)	
Non-Hispanic White	12 (75%)	17 (56.7%)	18 (78.3%)	48 (77.4%)	
**APOE e4, Positive, N (%)**	4 (26.7%)	4 (14.3%)	5 (22.7%)	36 (63.2%)	**< 0.001§**
**Diabetes, N (%)**	5 (31.2%)	13 (43.3%)	5 (21.7%)	10 (16.1%)	**0.040**
**Hypertension, N (%)**	14 (87.5%)	26 (86.7%)	20 (87.0%)	46 (74.2%)	0.386
**Hyperlipidemia, N (%)**	13 (81.2%)	23 (76.7%)	15 (65.2%)	45 (72.6%)	0.721
**Most Recent Assessment, N (%)**					
Not Demented	6 (37.5%)	13 (44.8%)	3 (13%)	1 (1.6%)	**< 0.001§**
Demented	6 (37.5%)	11 (37.9%)	16 (69.6%)	58 (93.5%)	
Mild Cognitive Impairment	4 (25%)	5 (17.2%)	4 (17.4%)	3 (4.8%)	
**Thal Amyloid Phase**					
A0 (Thal Phase 0)	16 (100%)	0 (0%)	0 (0%)	0 (0%)	**< 0.001**§
A1 (Thal Phase 1 or 2)	0 (0%)	26 (86.7%)	2 (8.7%)	0 (0%)	
A2 (Thal Phase 3), N (%)	0 (0%)	2 (6.7%)	11 (47.8%)	0 (0%)	
A3 (Thal Phase 4 or 5), N (%)	0 (0%)	2 (6.7%)	10 (43.5%)	62 (100%)	
**Braak NFT stage**					
B0 (Braak Stage 0), N (%)	2 (12.5%)	0 (0%)	0 (0%)	0 (0%)	**< 0.001**§
B1 (Braak Stage I or II), N (%)	9 (56.3%)	18 (60%)	0 (0%)	0 (0%)	
B2 (Braak Stage III or IV), N (%)	5 (31.3%)	12 (40%)	19 (82.6%)	0 (0%)	
B3 (Braak Stage V or VI), N (%)	0 (0%)	0 (0%)	4 (17.4%)	62 (100%)	
**CERAD score for density of neocortical neuritic plaques**					
C0 (No neuritic plaques)	16 (100%)	10 (33.3%)	0 (0%)	0 (0%)	**< 0.001**§
C1 (Sparse neuritic plaques)	0 (0%)	17 (56.7%)	3 (13.0%)	0 (0%)	
C2 (moderate neuritic plaques)	0 (0%)	3 (10%)	11 (47.8%)	14 (22.6%)	
C3 (frequent neuritic plaques)	0 (0%)	0 (0%)	9 (39.1%)	48 (77.4%)	

AD, Alzheimer disease; APOE e4, Apolipoprotein E4

£ ANOVA

† Chi-square test

§ Fisher exact test

### Immunohistochemistry

**Fig. 2:**
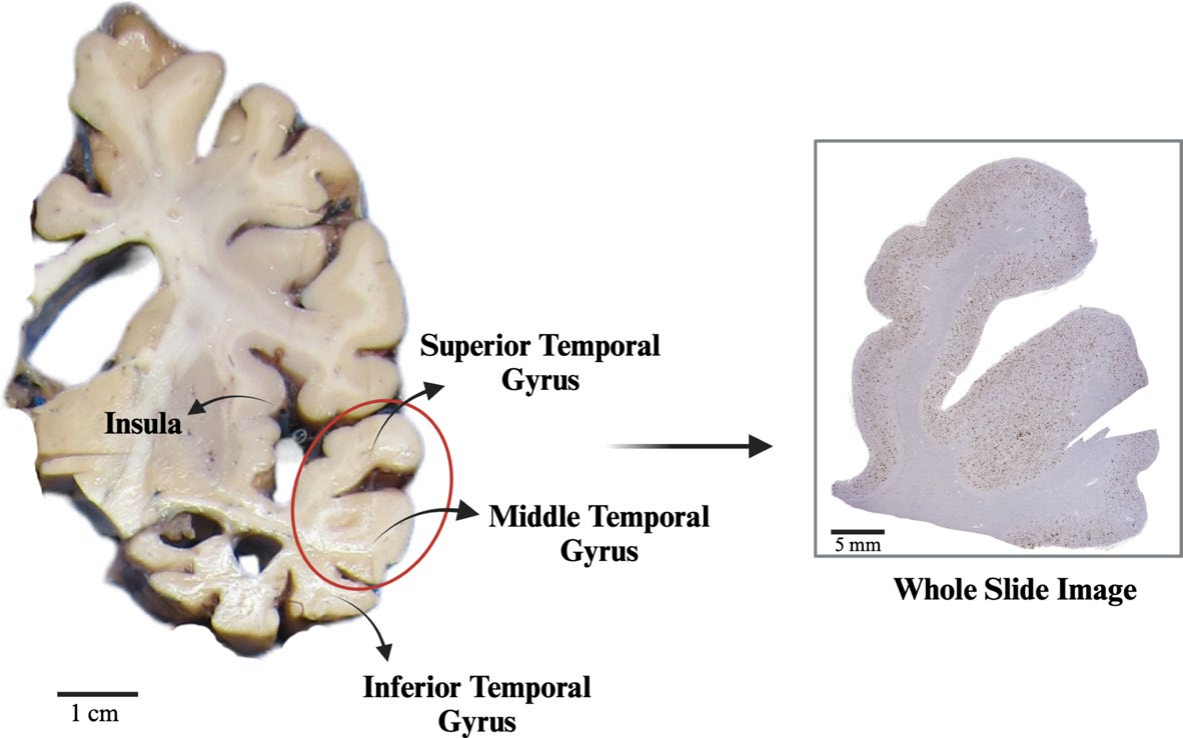
Illustrative diagram of temporal lobe sampling at the UC Davis ADRC Neuropathology Core. Coronal brain sections of the temporal gyri (middle and superior), approximately 1 cm thick, are obtained and sampled at the level of the insula and hippocampus. The histopathological analysis encompasses the superior and middle temporal gyri, which, after sampled, are paraffin-embedded, cut at 5 to 7 μm thick, mounted in glass slides, stained, and scanned at a 20x magnification, at a rate of 0.503 μm/pixel, to produce a Whole Slide Image that is generated as a CSV file and can be analyzed and annotated in the Aperio Image Scope software

We utilized standard histological coronal sections from 5 to 7 μm formalin-fixed paraffin-embedded (FFPE) sections derived from the temporal lobe (Fig. 2). All sections were deparaffinized through a graded series of alcohols treatments: unstained slides were imersed into two changes of 3 min each into Xylene (HistoPrep™—Fisher Scientific, Pittsburgh, PA, USA), followed by 2 changes of 100% alcohol (StatLab Medical Products, McKinney, TX, USA) for 2 min each, and then 2 changes of 95% alcohol for 2 min each. After deparaffinization, the slides were placed into distilled water. Slides then underwent pretreatment prior to staining including 10 min in 87% formic acid, endogenous peroxidases were block with 3% Hydrogen Peroxide with subsequent applications of primary and secondary antibodies. These sections underwent immunohistochemical labeling utilizing an Aβ-targeting antibody (1:1600 dilution; 4G8; Biolegend, San Diego, CA) and were subjected to color development using 3,3′-diaminobenzidine (DAB), followed by hematoxylin counterstain. Staining was conducted following standard procedures on automated machines (i.e. autostainers; DAKO AutostainerLink48, Agilent, Santa Clara, CA, USA) utilizing positive and negative controls. All immunhohistochemsitry staining procedures were performed at the UC Davis Histology Core, operating under the best laboratory practices standards and meeting Federal, State of California, and UC Davis guidelines and regulations.

### Slide digitization

Prior to scanning, glass slides containing the tissue were cleaned with a 70% ethanol solution. Scanning was conducted using an Aperio AT2 DX system at resolution of 0.503 micron per pixel. A small subset of 7 WSI were scanned at 0.252 micron per pixel. Prior to analysis, we used down sampling to homogenize the resolution of all WSIs to 0.503 micron per pixel, a magnification of 20x. Subsequently, all WSIs were uploaded to an on-premise server at UC Davis and accessed for viewing through a local instance of the Image Scope Software.

### Neuropathological evaluation

A neuropathological assessment was performed for each case during the evaluations, involving different experts and neuropathologists across multiple time points. The inclusion of relevant data, such as semi-quantitative scores of CERAD score, Thal Amyloid Phase, and Braak NFT stage [16, 20, 32], were collected using standardized forms from the National Alzheimer’s Coordinating Center (NACC), is presented in specific tables to provide a detailed description of the cohort. The study groups were defined based on NIA-AA criteria of Alzheimer Disease Neuropathological Changes (ADNC): No/Not ADNC, Low ADNC, Intermediate ADNC, and High ADNC [4].

### Clinical data

Available information regarding the presence of select clinical comorbidities was denoted based on data retrieved from NACC’s Uniform Dataset (UDS) [31]. Diabetes, hypertension, and/or hyperlipidemia were denoted as present if there was history of diagnosis (recorded within the UDS as active and/or inactive). Information regarding most recent assessment (MRA) was also retrieved from the UDS and clumped into three categories: Demented (for all cases denoted as demented); Mild Cognitive Impairment (MCI – for all cases denoted as Other cognitive impairment not meeting criteria for dementia); Not demented (for cases denoted as No cognitive impairment and Questionable cognitive impairment). Cases having MRA denoted as Diagnosis Deferred were not included in the analysis of clinical diagnosis (*n* = 1).

### Data preprocessing

Reinhard [33] color normalization was applied to all WSIs before analysis, using the same reference slide for all WSIs to ensure consistent color characteristics across different slides. Applying color normalization can minimizing batch effects; we have done previous works running the algorithm with and without Reinhard and there were no statistically significant differences with results [25]. The PyVips library [34] was used to implement the Reinhard normalization and tile the original WSIs into 1536 × 1536 pixel images uniformly. These tiles were then used as the input for our deep learning (DL) framework.

### Deep learning (DL) framework

Our DL framework includes two Convolutional Neural Network (CNN) models: one is for Aβ deposit classification denoted as *f*($\cdot$) [25]; the other is for GM/WM segmentation denoted as *g*($\cdot$) [27]. The Aβ deposit classification model was previously trained over 33,111 tiles at 256 × 256 pixel level to distinguish Aβ in the form of diffuse plaques, cored plaques, or cerebral amyloid angiopathy (CAA). Importantly, our segmentation excluded a classification of leptomeninges [27] and aligned with methodologies established in Tang et al. (2019) [25]. The weights of our CNN models were provided as previously published [25, 27] and loaded as pre-trained models to generate prediction maps. All CNN codes are implemented by Python’s open-source package PyTorch [35]. For the easy implementation on different platforms, we used Docker to build a Docker container to run all codes [36].

### Sliding window for inference

A sliding window method [37] was applied to visualize the distribution and location of Aβ pathologies from a global view by generating WSI heatmaps of predictions. These heatmaps plot the location of each deposit predicted by the CNN model*f*($\cdot$) up to the full WSI view. A stride size of 16 pixels was used to iterate through the WSI using the sliding window, resulting in the confidence heatmaps at a fraction of the resolution of original WSIs. This is helpful for the reproducibility without excessive loss of information when implemented on the modern devices equipped with an Intel Xeon 1U processor, 192GB of RAM, and a 16GB Nvidia Tesla T4 Graphic Processing Units (GPUs). For each slide, it would generate three confidence heatmaps corresponding to cored, diffuse, and CAA, separately. After, the cleaning and blob labeling were applied to the heatmaps, subsequently specific thresholds were applied to each Aβ deposit, which converted the heatmaps to binary masks: probabilities below the threshold would be converted to zero and above would be one.

To study the distribution of different Aβ deposits in GM and WM, we incorporated a previously published CNN model*g*($\cdot$) [27] into our system to generate prediction heatmaps of GM and WM by using the same sliding window of stride size at 16 pixels to guarantee the output of*f*($\cdot$) is of the same size of the output of*g*($\cdot$) so that they can be pixel-wise overlapping.*g*($\cdot$) was based on ResNet-18, a widely used CNN architecture, by modifying the last fully connected layer to output the possibility of three categories: GM, WM, and background.

The heatmap of GM/WM was overlaid with the Aβ deposits’ heatmap as shown in Fig. 7: cyan denotes GM, yellow denotes WM, and orange denotes Aβ-deposits. We visualized the distribution of each type of Aβ-deposits, separately. The average time to process each WSI with the entire workflow on a 16GB Nvidia Tesla T4 is 6 h.

### Aβ-deposits counting

**Fig. 3:**
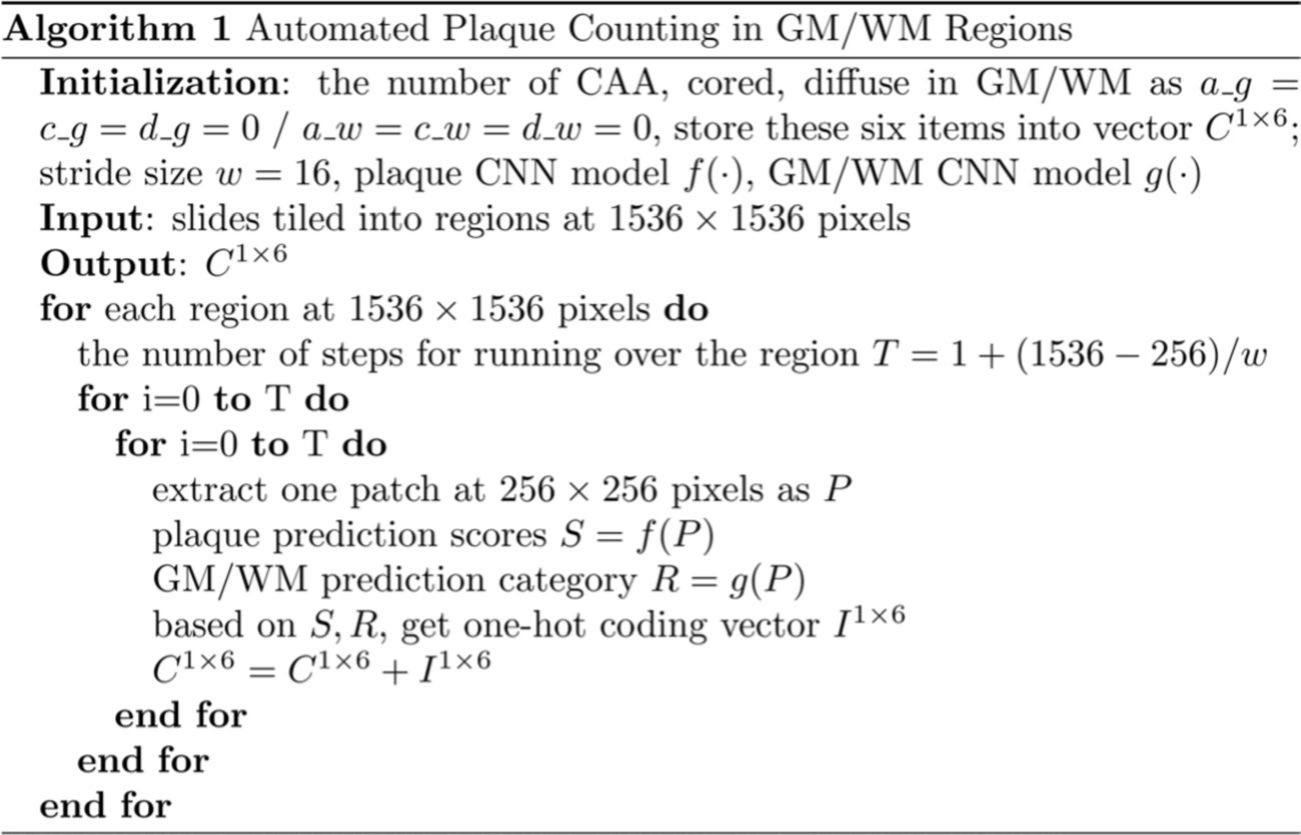
Overview of the convolutional neural network (CNN) machine learning counting algorithm for automated core and diffuse plaque and cerebral amyloid angiopathy (CAA) counting in grey (GM) and white (WM) regions

The Aβ-deposits counting algorithm is summarized in Fig. 3. A complete description of the algorithm was previously published by our group [25, 27]. A zero vector *P* with the shape of 1 × 6 is set up to record the number of each type of Aβ deposit (CAA, cored, diffuse) in GM and WM regions separately as the initialization. Then, each slide would be normalized and tiled into 1536 × 1536 pixel images. After, the sliding window was applied to extract a patch *P* at 256 × 256 pixels in order from left to right, top to bottom. The patch would be the input of CNN models *f*($\cdot$) and *g*($\cdot$) to output the predictions in terms of the type of Aβ deposit and the region category. These predictions were converted into a temporary one-hot vector *I*, which was added to *I* to update *C* until the sliding window walked over the whole tissue slide. The final output is the vector *C* containing all counting information of each type of Aβ-deposit in GM and WM.

### Statistical analysis

Extracted data were assessed for normality using histograms, Q-Q plots, and the Shapiro-Wilk test. Demographic, clinical, and neuropathologic characteristics are summarized separately according to the ADNC group (Table 1). Quantitative variables are summarized by means and standard deviation, and the means across groups were compared by ANOVA. Categorical variables were presented as frequencies and percentages and compared across groups by the Chi-square test or Fisher’s exact test. CNN-derived variables related to Aβ-deposits and CAA are presented as median with interquartile range (IQR). Kruskal-Wallis was used to compare these variables across groups, followed by post-hoc pairwise comparisons using Dunn’s test with the Bonferroni correction for multiple comparisons to identify specific differences. When only two groups were compared, Wilcoxon rank sum test was used. Analyses of individual variables were restricted to decedents with non-missing data, with no attempt at imputation. Statistical significance was set at *P* value < 0.05; multiple comparison adjusted p-values are reported when appropriate. All statistical tests were performed using R, RStudio (R version 4.2.3) and the tidyverse package [38]. Figures were designed using Biorender and RStudio package ggplot2 [39].

## Results

### Demographics and clinical data

A total of 131 persons with available WSI of the temporal lobe (decedents with completed neuropathology reports between December/2012 to October/2019) were included in our analyses. Cohort demographics, neuropathologic, and clinical characteristics of all participants and ADNC groups are presented in Table 1. With respect to gender, 48.9% of our cohort were females. Age at death (*P* = 0.31) and gender (*P* = 0.89) distributions, as well as formal education attainment (*P* = 0.06), were not significantly different across ADNC groups. In our cohort, the High ADNC group had 63.2% of individuals having at least one APOE ε4 allele (*P* < 0.001). Some differences were found with clinical comorbidities, with decedents in the Low ADNC group having the highest rate of diabetes (*P* = 0.04). Although the percentages of hypertension and hyperlipidemia were not significant between groups (*P* = 0.39 and *P* = 0.72, respectively), they were both present in over 60% of individuals in each group. Regarding the most recent assessment (MRA), 23 cases were categorized as Not Demented (17.7%), 16 cases as mild cognitive impairment (MCI), and 91 cases were categorized as demented, with the majority of the demented cases (63.7%), in the High ADNC group (*P* < 0.001).

### Aβ deposits count by ADNC groups

**Fig. 4:**
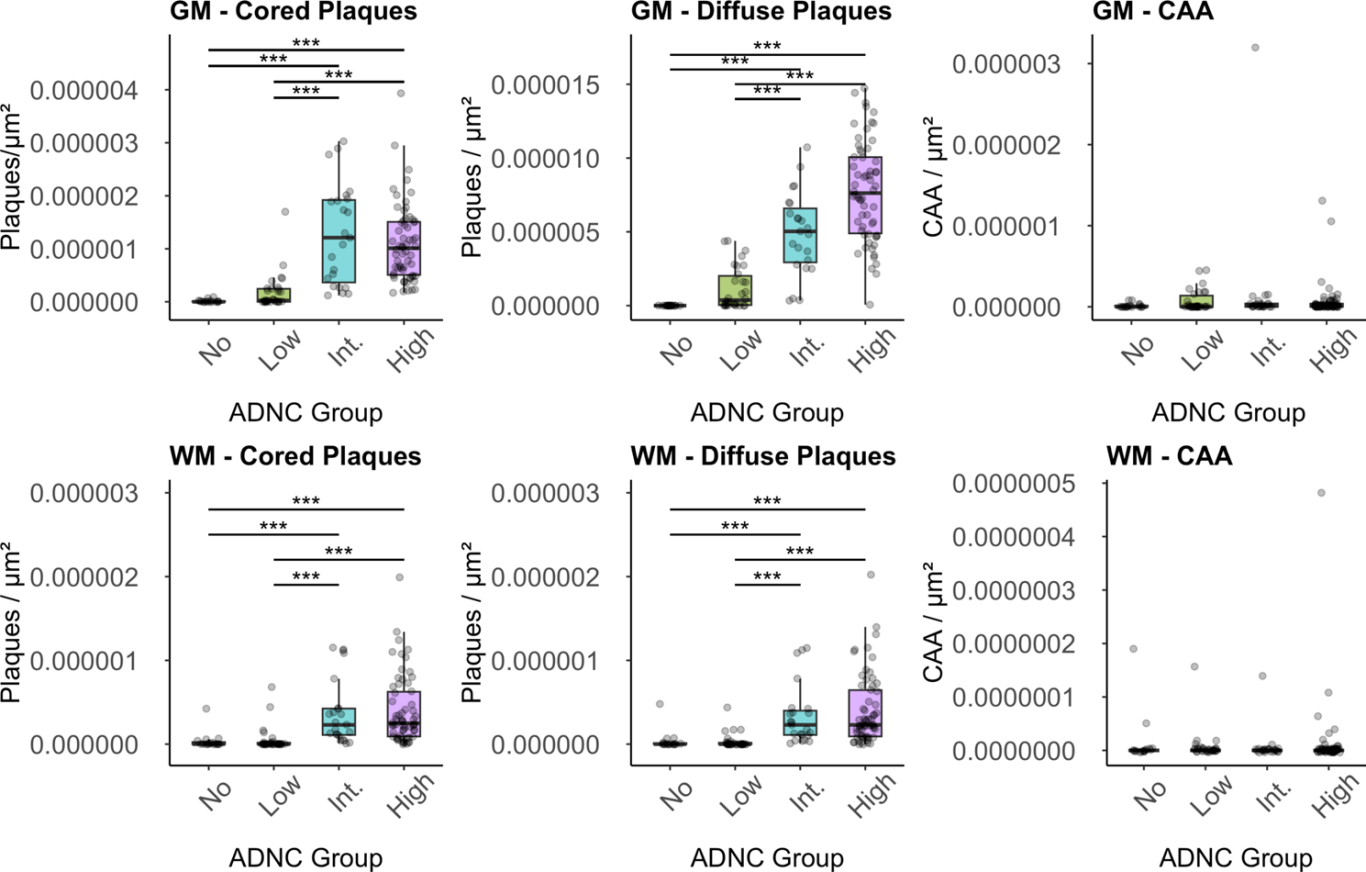
The boxplots depict the median Aβ deposits in the temporal lobe grey matter (GM- top panels) or white matter (WM-bottom panels), stratified by their assignments to Alzheimer Disease Neuropathologic Change (ADNC) of No = Not, Low, Intermediate = Int., or High. The horizontal line marks the median, the box encapsulates the interquartile range (IQR), and the whiskers extend to the smallest and largest observation within 1.5 times the IQR of the bottom and top of the box (* = *p* < 0.05; ** = *p* < 0.01; and *** = *p* < 0.001)

Aβ deposits, categorized by their ADNC groups assignments for the UC Davis dataset, are depicted in Fig. 4 and summarized in Table 2.

**Table 2:** Count of plaques and cerebral amyloid angiopathy (CAA) by Alzheimer’s Disease Neuropathologic Change (ADNC)

	No/Not ADNC (*n* = 16)	Low ADNC (*n* = 30)	Intermediate ADNC (*n* = 23)	High ADNC (*n* = 62)	*P* value
**Gray Matter**					
Cored Plaques, raw count, median (CI)	0.5 (0, 4)	6 (1, 38)	283 (118, 484)	265 (182, 333)	**< 0.001**
Diffuse Plaques, raw count, median (CI)	0 (0,0)	74.5 (25, 279)	1358 (610, 2134)	1998 (1764, 2433)	**< 0.001**
CAA, raw count, median (CI)	0 (0, 11)	1 (0, 10)	5 (0, 11)	3 (2, 5)	0.055
Cored Plaques/µm², median (CI)	0.00000000 (0.00000000, 0.00000002)	0.000000025 (0.000000010, 0.000000210)	0.00000121 (0.00000044, 0.00000190)	0.00000101 (0.00000068, 0.00000120)	**< 0.001**
Diffuse Plaques/µm², median (CI)	0 (0, 0)	0.000000375 (0.000000090, 0.000001620)	0.00000503 (0.00000309, 0.00000624)	0.000007625 (0.000006140, 0.000008860)	**< 0.001**
CAA/µm², median (CI)	0.00000000 (0.00000000, 0.00000003)	0.00000000 (0.00000000, 0.00000002)	0.00000001 (0.00000000, 0.00000004)	0.00000001 (0.00000001, 0.00000002)	0.182
**White Matter**					
Cored Plaques, raw count, median (CI)	0 (0, 2)	0 (0, 0)	11 (6, 27)	17 (12, 22)	**< 0.001**
Diffuse Plaques, raw count, median (CI)	0 (0, 0)	0 (0, 1)	5 (3, 31)	18.5 (13, 25)	**< 0.001**
CAA, raw count, median (CI)	0 (0, 0)	0 (0, 0)	0 (0, 0)	0 (0, 0)	0.89
Cored Plaques/µm², median (CI)	0.00000000 (0.00000000, 0.00000004)	0 (0, 0)	0.00000023 (0.00000012, 0.00000042)	0.00000025 (0.00000020, 0.00000035)	**< 0.001**
Diffuse Plaques/µm², median (CI)	0.00000000 (0.00000000, 0.00000002)	0 (0, 0)	0.00000023 (0.00000012, 0.00000038)	0.00000023 (0.00000019, 0.00000035)	**< 0.001**
CAA/µm², median (CI)	0 (0, 0)	0 (0, 0)	0 (0, 0)	0 (0, 0)	0.88

### Gray Matter vs. white matter

Median levels of cored plaques/µm² in GM were highest in the Intermediate ADNC group, followed by High ADNC compared to the Low/Not ADNC. Post-hoc analysis confirmed statistical significance between adjacent (Low vs. Intermediate ADNC) and non-adjacent groups (Low vs. High ADNC, No/Not vs. Intermediate ADNC, and No/Not vs. High ADNC groups), all yielding a P-value < 0.001. Regarding GM diffuse plaques/µm², similar trends were shown, with statistical significance observed between adjacent groups (Low vs. Intermediate ADNC, *P* = 0.001), and a suggestive trend between Intermediate and High ADNC (*P* = 0.055). Non-adjacent group comparisons also demonstrated significant differences (No/Not vs. Intermediate ADNC, No/Not vs. High ADNC, and Low vs. High ADNC; *P* < 0.001). Notably, CAA/µm² showed very small values across all groups, with multiple high-score outliers for the High ADNC group and one in the Intermediate ADNC group; however, there were no significant differences across the groups.

In the analysis of Aβ-deposits within WM, the median levels of cored plaques exhibited a progressive increase in plaques per unit area (µm²) in correspondence with escalating severity of the ADNC group (No/Not, Low, Intermediate, and High) in Table 2. Post-hoc analysis unveiled statistical significance between the adjacent groups Low and Intermediate ADNC (*P* < 0.001), alongside significant differences among non-adjacent groups (No vs. Intermediate, No/Not vs. High, Low and High ADNC groups) (*P* < 0.001). Parallel patterns were evident in the assessment of diffuse plaques within the WM, with significant differences noted between the adjacent groups Low and Intermediate ADNC (*P* < 0.001), as well as between non-adjacent groups (No/Not vs. Intermediate, No/Not vs. High, Low vs. High ADNC groups) (*P* < 0.001). Regarding WM CAA, notably low values were observed across all groups, without discernible significant differences.

### Aβ deposits counts grouped by ABC score (NIA Regan Criteria)

**Fig. 5:**
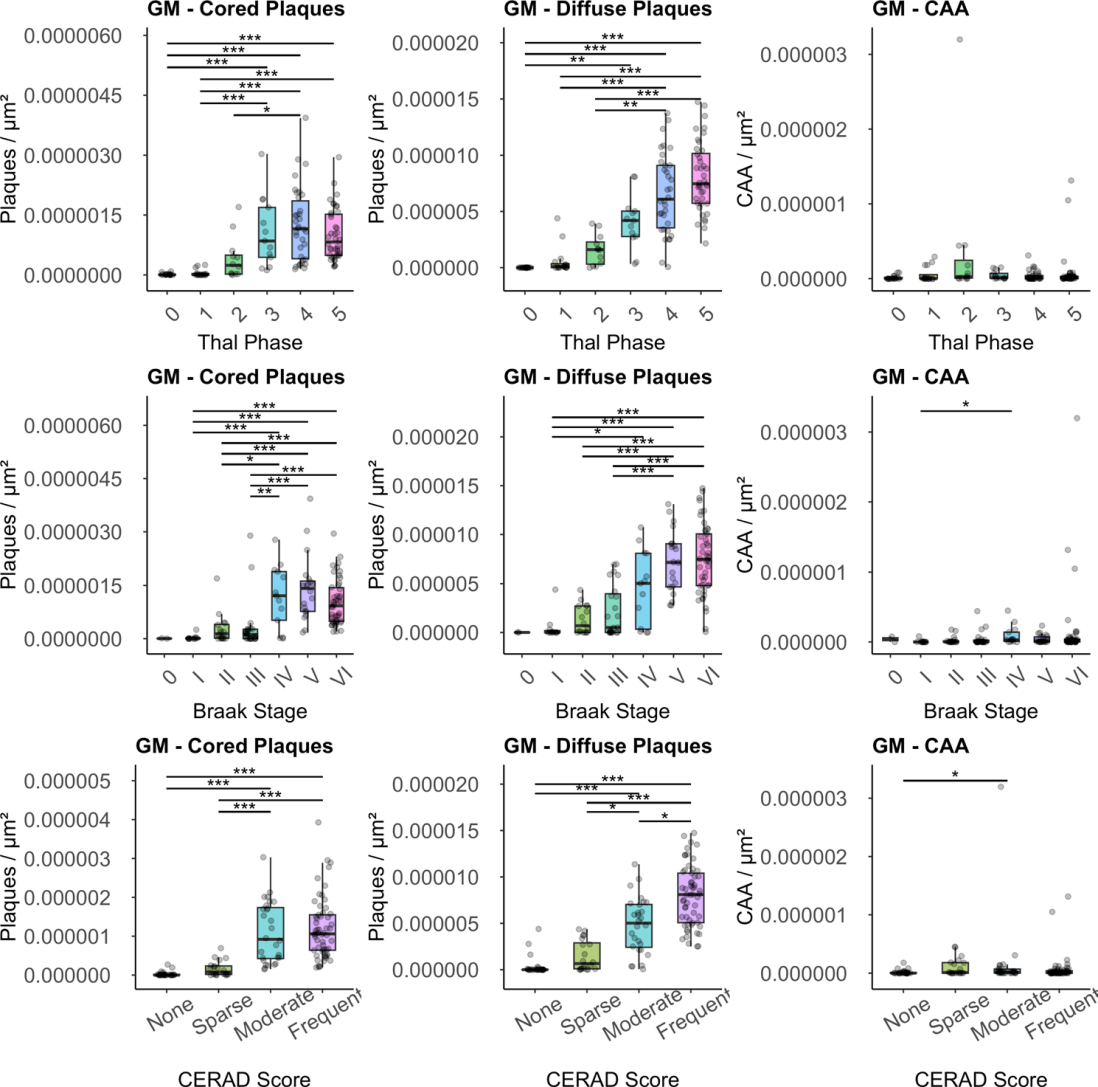
The boxplots depict the median Aβ deposits in the gray matter (GM), stratified by their assignments according to staging/phases (Thal Phase = Thal Amyloid Phase, Braak Stage = Braak Neurofibrillary Tangle Stage and CERAD score = CERAD Neuritic Plaque score). The horizontal line marks the median, the box encapsulates the interquartile range (IQR), and the whiskers extend to the smallest and largest observation within 1.5 times the IQR of the bottom and top of the box (* = *p* < 0.05; ** = *p* < 0.01; and *** = *p* < 0.001)

An alternative approach employed in our study involved categorizing AD cases based on the semi-quantitative staging scales utilized to define likelihood of AD by NIA-AA ADNC criteria. The counts of Aβ deposits in GM, grouped according to the NIA staging scales, are depicted in Fig. 5 (for WM in Supplemental Material, Fig S1).

### A – Thal Amyloid Phase

In the analysis of GM cored plaques in the temporal lobe, a notable upward trend in median cored plaque density (plaques/µm²) was observed alongside the progression of Thal Amyloid Phase (Thal Phases 0, 1, 2, 3, 4 and, 5), demonstrating a statistically significant trend (*P* < 0.001). Subsequent post-hoc analysis revealed statistical significance between non-adjacent groups: Phase 0 vs. Phase 3, Phase 1 vs. Phase 3, Phase 0 vs. Phase 4, Phase 1 vs. Phase 4, Phase 0 vs. Phase 5, Phase 1 vs. Phase 5 (all with *P* < 0.001), and Phase 2 vs. Phase 4 (*P* = 0.024). Regarding GM diffuse plaques/µm², similar trends were observed, with statistical significance observed between non-adjacent groups: Phase 0 vs. Phase 4; Phase 1 vs. Phase 4; Phase 0 vs. Phase 5; Phase 1 vs. Phase 5, and Phase 2 vs. Phase 5 (all with *P* < 0.001) and Phase 0 vs. Phase 3 (*P* < 0.002) and Phase 2 vs. Phase 4 (*P* < 0.004). GM CAA/µm² yielded minimal values across all groups, with high-score outliers for groups Phase 2 and 5; however, these findings did not attain statistical significance.

For WM analysis of Aβ-deposits, a similar trend was observed for the median levels of both cored and diffuse plaques, with a progressive increase in plaques per unit area (µm²) in correspondence with escalating severity of the Thal Amyloid Phase. Post-hoc analysis unveiled statistical significance for cored and diffuse plaques, between the non-adjacent groups: Phase 0 vs. Phase 4; Phase 0 vs. Phase 5; Phase 1 vs. Phase 3; Phase 1 vs. Phase 4; Phase 1 vs. Phase 5 (all with *P* < 0.001); Phase 0 vs. Phase 3 and Phase 1 vs. Phase 3 (both with *P* < 0.02); Phase 2 vs. Phase 5 (*P* < 0.05; Cored plaques only). Regarding WM CAA, low values were observed across all groups, and there were statistically significant differences across groups (Supplemental Material, Fig S1).

### B – Braak NFT Stage

The analysis of both GM cored and GM diffuse plaques in the temporal lobe indicated a substantial rise in the median values of plaques/µm², correlating with the progressive of Braak NFT stage (*P* < 0.001). The adjacent Braak NFT stages III vs. IV (*P* = 0.003) showed statistical significance for temporal lobe cored plaques. Additionally, differences emerged among non-adjacent Braak NFT stages II vs. IV (*P* = 0.01); I vs. IV, I vs. V, II vs. V, III vs. V, I vs. VI, II vs. VI, and III vs. VI (all with *P* < 0.001). The analysis for temporal lobe diffuse plaques followed an analogous pattern, with statistical significance observed only between non-adjacent Braak NFT stages I vs. V, II vs. V, III vs. V, I vs. VI, II vs. VI, and Braak NFT stages III vs. VI (all with *P* < 0.001), and Braak NFT stages I vs. IV (*P* < 0.05). Analysis of temporal lobe CAA/µm² also presented marginal values yet displayed statistical significance between non-adjacent Braak NFT stages (I vs. IV (*P* = 0.02)).

While analyzing deposits in the WM, a similar pattern was encountered for the median levels of both cored and diffuse plaques, with a progressive increase in plaques per unit area (µm²) in correspondence with escalating severity of Braak NFT stage. A statistical significance was observed after post-hoc analysis, for both cored and diffuse plaques, between the non-adjacent Braak NFT stages: I vs. IV (*P* < 0.005); II vs. V and III vs. V (*P* < 0.01); and I vs. V; I vs. VI; II vs. VI; III vs. VI (all with *P* < 0.001). The analysis of WM CAA indicated notably low values, but no significant differences across Braak NFT stages (Supplemental Material, Fig S1).

### C- CERAD score

The analysis of both temporal lobe GM cored and GM diffuse plaques indicated a significant increase in the median values of plaques/µm² in a progressive manner, evident in tandem with the escalation in severity of CERAD neuritic plaque score (none, sparse, moderate, and frequent) (*P* < 0.001). For cored plaques, the adjacent groups sparse vs. moderate showed statistical significance (*P* < 0.001). Moreover, differences were observed among non-adjacent groups (none vs. frequent, none vs. moderate, and sparse vs. frequent) (*P* < 0.001). For diffuse plaques, the same differences were found with the addition of moderate vs. frequent (*P* < 0.02) and a reduced difference for sparse vs. moderate (*P* < 0.02). Significant differences were also observed for CAA/µm², even with small values, between non-adjacent groups none vs. moderate *P* < 0.05).

In the WM, for both cored and diffuse plaques, differences were observed among adjacent groups sparse vs. moderate (*P* < 0.001) and for non-adjacent groups (none vs. moderate; none vs. frequent; sparse vs. frequent) (*P* < 0.001). No significant differences were observed in the analysis of WM CAA (Supplemental Material, Fig S1).

### Aβ deposits count grouped by APOE ε4 carriers

**Fig. 6:**
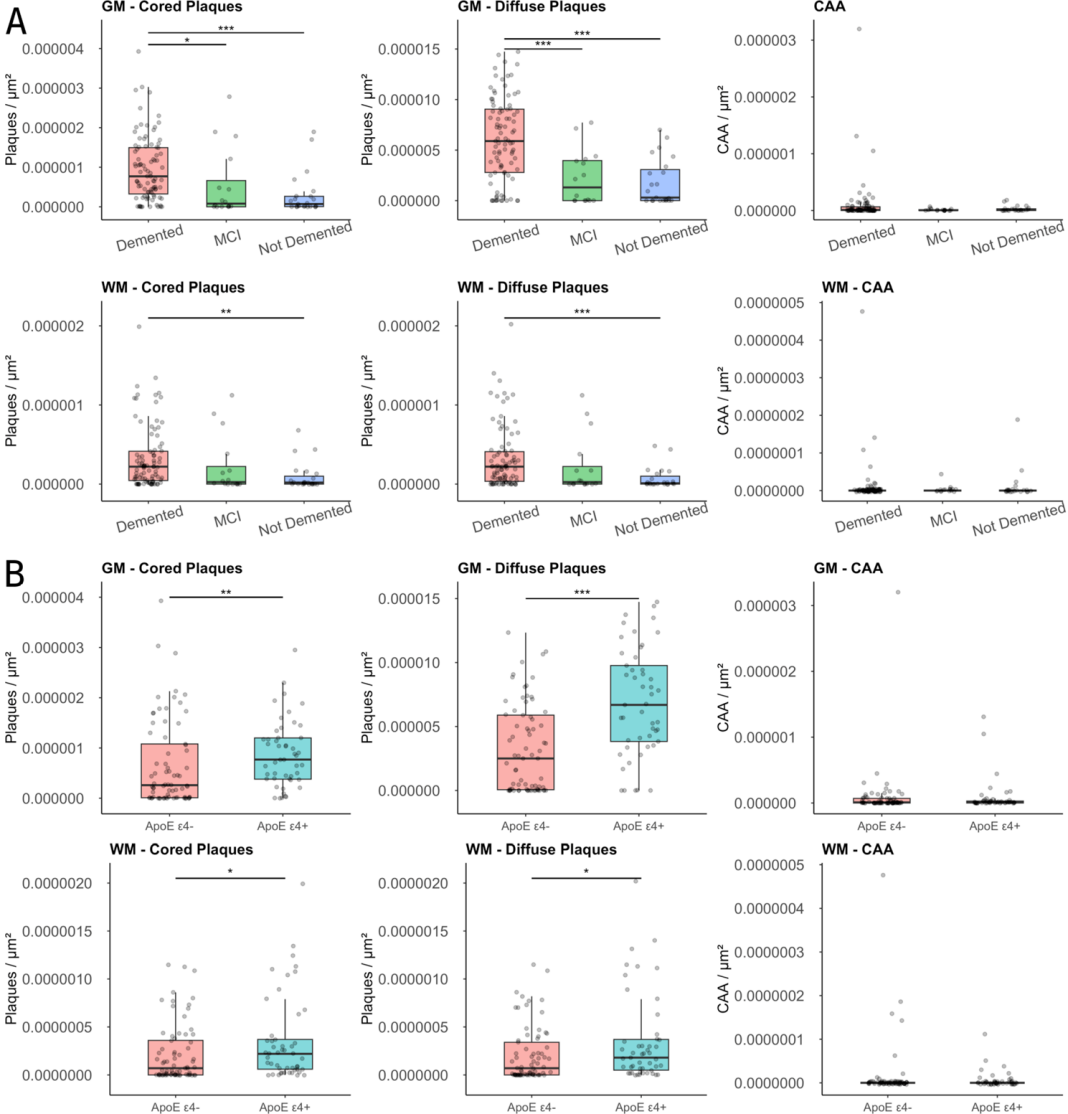
Aβ deposits densities in grey (GM) and white matter (WM) grouped based on genetic (presence or absence of an APOE Ɛ4) and clinical diagnostic group (Demented, MCI = mild cognitive impairment, or Not Demented**). A**) Boxplots depict the median of Aβ deposits, illustrating the impact of APOE E4 allele presence within the investigated cohort. The top row illustrates the anterior distribution of parameters within GM, while the bottom row showcases deposits in WM. The horizontal line marks the median, the box encapsulates the interquartile range (IQR), and the whiskers extend to the smallest and largest observation within 1.5 times the IQR of the bottom and top of the box. **B**) Boxplots depict the median Aβ deposits in the Gray Matter, stratified by their assignments according to Most Recent Assessment. The horizontal line marks the median, the box encapsulates the interquartile range (IQR), and the whiskers extend to the smallest and largest observation within 1.5 times the IQR of the bottom and top of the box (* = *p* < 0.05; ** = *p* < 0.01; and *** = *p* < 0.001)

ApoE ε4 genotyping information was available for 93.1% of the cases in our cohort (*n* = 122). Among these, 40.2% (*n* = 49) were classified as ApoE ε4 allele carriers (ApoE ε4+). The remaining 59.8% of cases (*n* = 73) were categorized as non-carriers (ApoE ε4-). Female/male ratios (*P* = 0.82) and formal education attainment (*P* = 0.98) were not significantly different between carriers and non-carriers; therefore, no adjustments in the analyses were made. ApoE ε4 + carriers exhibited higher GM cored plaques/µm² levels compared to the ApoE ε4- group (*P* = 0.007, Fig. 6).

Similarly, temporal lobe GM diffuse plaques/µm² values were significantly higher in the ApoE ε4 + group than in the ApoE ε4- group (*P* < 0.001). Median CAA/µm² values displayed minimal variation between groups and lacked statistical significance.

Analysis of Aβ-deposits in the temporal lobe WM revealed analogous patterns, with cored plaques/µm² displaying higher levels in the ApoE ε4 + group compared to the ApoE ε4- group (*P* = 0.02). Likewise, temporal lobe WM diffuse plaques/µm² values were approximately 1.5 times higher in the ApoE ε4 + group (*P* = 0.02). Median WM CAA/µm² values also demonstrated minimal differences between groups and lacked statistical significance (Fig. 6).

### Aβ deposits count grouped by most recent assessment (MRA)

Data from the most recent assessment (MRA) were available for 130 cases, with only one case classified as diagnosis deferred. Among these cases, 70% (*n* = 91) were diagnosed as demented, 17.6% (*n* = 23) as not demented, and 12.3% (*n* = 16) as MCI. No significant differences in age (*P* = 0.55) and educational attainment (*P* = 0.82) were observed among these groups. However, a statistically significant difference was found in female/male ratios (chi-squared test, *P* = 0.005) with a higher percentage of females in the not demented (78.3%) compared to the MCI (31.2%) and demented (45%) groups. Hence, no adjustments were made for age, sex, or educational attainment in subsequent analyses.

When analyzing the differences in GM Aβ-deposits, significant differences were observed in the count of cored plaques and diffuse plaques / µm² between groups (*P* < 0.001; Fig. 6). The median count of GM cored plaques was higher in the demented then in the MCI (*P* < 0.02) and not demented (*P* < 0.001) groups. Regarding diffuse plaques, significant differences in median counts were observed between the demented and MCI (*P* < 0.001) and not demented (*P* < 0.001) groups. When examining WM plaques, significant differences were found between the demented and not demented groups for both cored (*P* = 0.001) and diffuse plaques (*P* = 0.004). No differences in CAA counts were observed between groups for both GM (*P* = 0.45) and WM (0.78) in our cohort (Fig. 6).

### Aβ deposits count grouped by mixed pathologies

The pathological diagnoses for each case are detailed in Supplemental Table 1. For this analysis, any case missing an evaluation of any of the three pathologies (AD, LBD, TDP-43) were removed from their respective category. Based on the collected data, three cohort subsets were identified: cases with no/minimal AD pathology (Not/Low ADNC group, Thal Phase and CERAD Score = 0, and plaque count = 0) were classified as control group (non-AD brain, *n* = 7), pure AD (no additional diagnosis-i.e. no Lewy body (LB) or TDP inclusions, *n* = 16), and all AD (any case with an ADNC of intermediate/high regardless of present/absence of LBs or TDP deposits, *n* = 85). The AD groups can be further broken down into pure AD group (*n* = 16), AD + LBD group (*n* = 28), and the AD + TDP group (*n* = 29); the LBD and TDP groups are not mutually exclusive as there are 16 individuals with AD + TDP + LBD. In addition, there were 28 cases with AD pathology but who were missing either the LB or TDP-43 data. Comparative analysis of these groups with regard to cored and diffuse plaque counts in the GM is illustrated in the Supplemental material, Fig. S2, S3, S4. Notably, statistical significance was observed between the pure AD and control groups, as well as between the all AD group and the control, determined through a Wilcoxon rank-sum test (Fig. S2). The comparison among the pure AD, AD + TDP, and AD + LBD groups (those with all three pathologies were removed to assure groups were independent) was performed with a Kruskal-Wallis test, revealing no significant differences among the groups in terms of cored and diffuse plaques (Supplemental Material, Fig. S3). Furthermore, the CAA/µm² median values in the temporal lobe exhibited no significant differences in any of these comparative analyses.

As a secondary exploratory analysis, we compared the medians of cored and diffuse plaques/ µm² in GM between two other cohort subsets: cases with and without the presence of LB inclusions (LBD group and no LBD group) and cases with and without the presence of TDP-43 inclusions (TDP-43 group and no TDP-43 group), regardless of ADNC group. For the LBD vs. no LBD groups, a trend was observed in the count of cored plaques/ µm² (*P* = 0.11), and a significance was observed between groups in the count of diffuse plaques/ µm² (*P* = 0.01). No differences were observed among the TDP-43 and no TDP-43 groups (Fig. S4).

### Total plaque distribution

**Fig. 7:**
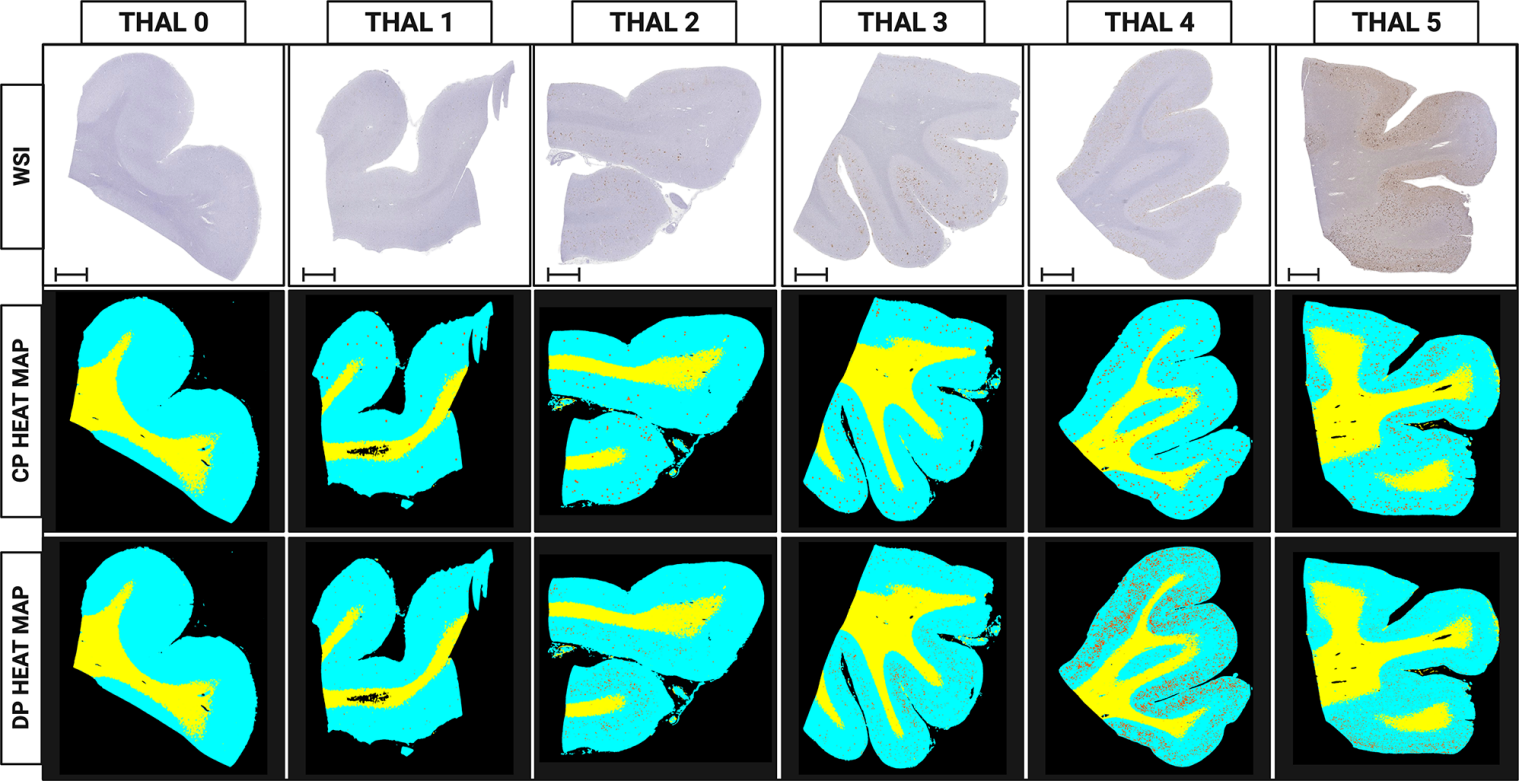
Examples of Aβ plaques in the temporal lobe by Thal Amyloid Phase seen in whole slide images of Aß immunohistochemically stained slides (top row) and heat map with the cored plaques (CP, middle row) and diffuse plaques (DP, bottom row) counting generated by the algorithm. Black is background, Cyan represents gray matter (GM), yellow represents white matter (WM) and orange dots represent individual plaques identified; Bar = 5 mm (* = *p* < 0.05; ** = *p* < 0.01; and *** = *p* < 0.001)

Our methodology was efficient in the segmentation of GM/WM and in the quantification as well as morphology classification of Aβ deposits (Heatmap examples in Fig. 7). The heatmap displays the mask generated by the algorithm, which highlights the locations of Aβ deposits (denoted in orange), while grey matter and white matter are denoted in cyan and yellow, respectively. This mask is created through the sliding window approach, where the algorithm iteratively processes patches of the whole-slide image, classifying each patch to generate a comprehensive map of deposit locations, enabling to observe the spatial distribution of the of Aβ deposits in the temporal lobe. There was a significant difference in the median of diffuse plaques (1164; 78-2134) and cored plaques (142; 21.5, 330) in the GM (*P* < 0.001). No significant difference was observed in the counts of diffuse and cored plaques in the WM. (Supplementary Data – Fig S1) There were significantly more plaques in the GM than the WM, for both cored and diffuse plaques. Median values for CAA were higher in the GM than in the WM (*P* < 0.001). Comparisons between groups were performed using the Wilcoxon rank sum test.

## Discussion

In the present study, we took a multi-disciplinary approach involving ML engineers, statisticians, clinicians, and neuropathology experts to validate a comprehensive workflow for the quantification of three distinct morphologic types of Aβ deposits—cored plaques, diffuse plaques, and CAA—in WSIs obtained from the temporal lobe of 131 cases. Additionally, we introduce an automated segmentation methodology for the delineation of GM and WM regions, enhancing the specificity of this method. Our investigation substantiates the robustness of the CNN pipeline, directly applying the model to a new cohort and showcasing its proficiency akin to experts. Our results reveal a compelling association between the outcomes derived from our quantitative methodology utilizing only one 5–7 μm section of a single brain region and the increase in severity observed across commonly utilized semi-quantitative staging scales, further validating the efficacy of our approach compared to established diagnostic schematics.

The current criteria for staging ADNC, as defined by the NIA-AA, offers a robust framework for assessing the progression of AD pathology. These criteria have demonstrated reproducibility and exhibit a strong correlation with the clinical manifestation of dementia [4]. Although these semi-quantitative approaches may be susceptible to variations in interpretation among different raters [16, 20–22, 32], it still provides a reliable and systematic approach. Importantly, these schematics have played a significant role in establishing crucial milestones within the field of dementia research and diagnosis [10, 40].

The evolution of WSI technologies and imaging analysis software ushered in the era of digital pathology. This advancement has made quantitative analyses more feasible and holds the potential to address the limitations of semi-quantitative scoring systems [28]. In our study, which focused on evaluating a specific brain region (WSIs of the temporal lobe), our findings align closely with the NIA-AA criteria. Notably, all cases in the High ADNC group exhibited advanced stages according to the NIA criteria, including Thal Amyloid Phase 4 and 5, Braak NFT Stage V or VI, and CERAD neuritic plaque score of moderate or frequent. Furthermore, a significant majority of individuals with dementia also fell into the High ADNC group. This underscores the importance of integrating semi-quantitative diagnostic approaches with quantitative analyses in comprehensively understanding AD pathology.

Our data were congruent with a similar ML pipeline; Vizcarra et al. [28] identified a correlation between CNN scores and CERAD-like scores. We encountered notable trends in our analysis of CAA. Our data also exhibited very low CAA values, with the presence of high-score outliers. This phenomenon may be attributed to the challenge of leptomeninges during the GM/WM segmentation process, potentially influencing the final CAA count as the only segmentation categories were GM, WM, and background. Future works evaluating vessels as cortical or leptomeningeal with CAA pathology may provide more relevant/accurate data, as well as denoting CAA within capillaries. Interestingly, our investigation into the compounding effects of mixed pathologies on Aβ deposition led to intriguing observations. In alignment with Vizcarra et al., we identified a significant difference in plaque counts when comparing cases with pure AD pathology to normal controls. However, this contrasted with the counts of plaques in individuals with concurrent deposits (AD + TDP-43, AD + LBD, or AD + LBD + TDP-43). Recent studies have elucidated the complex nature of AD pathology and its correlations with mixed pathologies, posing a significant challenge for diagnosis, prognosis, and treatment strategies [41–43]. Other research groups have also demonstrated the effectiveness of ML pipelines in quantifying Tau pathology [44–48]. These results collectively contribute to our understanding of AD pathology and the potential impact of mixed pathologies on quantitative assessments, further highlighting the importance of leveraging ML techniques for comprehensive analyses.

The apolipoprotein E (APOE) gene, located on chromosome 19, ranks among the most potent and widespread genetic risk factors for AD, impacting over half of the cases [49–51]. APOE ε4 allele carriers, about 23% of the world population [52], not only exhibit a heightened susceptibility to AD but it also can actuate the presence of co-pathologies across various conditions [53, 54]. The influence of a single APOE ε4 allele on the magnitude and severity and the regional influence of this gene on Aβ-related pathology has been demonstrated [55]. Our findings closely align with the established understanding of APOE ε4 pivotal role in driving Aβ accumulation. Within our cohort, ApoE ε4 carriers (37.4%) demonstrated almost two-fold higher GM cored plaques/µm² levels (*P* = 0.02) and 1.5-fold more GM diffuse plaques/µm² (*P* < 0.01). A parallel trend emerged in the analysis of Aβ-deposits in the WM among the ApoE ε4 + group, wherein cored plaques/µm² exhibited a twofold increase (*P* = 0.01), and WM diffuse plaques/µm² values were approximately 1.5 times elevated (*P* < 0.01). These findings further underscore the clinical relevance of APOE genotyping as a potential prognostic marker in AD. The high APOE ε4 prevalence within the High ADNC group echoes its established correlation with disease progression, endorsing the utility of a harmonized quantitative approach for profiling Aβ deposits in tandem with genetic information.

Aβ deposits can be observed in the human brain in a wide array of morphologies, which are potentially linked to specific clinical features [13, 56]. Among these forms, diffuse plaques are thought to represent the earliest and most prevalent manifestation of Aβ deposition, constituting over 50% of the total plaque burden [40, 57, 58]. Our study substantiates existing knowledge by revealing substantial differences in the raw counts of both diffuse and cored plaques [40]. While Aβ pathology primarily manifests in GM, it is worth recognizing plaque presence has also been documented in the WM [17], a phenomenon substantiated by our findings. Notably, our results confirm a lower rate of Aβ deposition in WM when compared to GM, even encompassing cerebral amyloid angiopathy (CAA) pathology (Supplemental Data – Fig S1). With the WM plaques, examining heatmaps, they were typically found in proximity to the boundary of GM/WM (vary rarely within deeper WM regions). As these data were from WSIs, a 2-dimensional representation of a 3-dimensional object, WM plaques may be manifested within GM on adjacent sections. Additional research having serial sectioning would aid in determining. The spatial distribution of Aβ aggregates in the human brain follows a structured hierarchical progression, as initially described by Thal and colleagues [16]. This cascade typically commences in the neocortex, subsequently extending into limbic structures, the diencephalon, basal ganglia, and finally reaching the brainstem and cerebellum. Of particular significance, our data suggest most demented cases align with Thal Amyloid Phases 4 and 5, whereas lower Thal Phases are more commonly observed in asymptomatic individuals. This observation confirms the utility of Thal Phases as a marker for disease progression and clinical manifestation in the context of AD pathology (See Figs. 5 and 7).

The intricate and multifactorial nature of AD pathogenesis requires a global task-force commitment to leverage innovative technologies for advancing the comprehension of disease mechanisms, heterogeneity, and strategies for early detection and progression prevention [10]. It is important to acknowledge the multidisciplinary nature of this study, which is increasingly vital for addressing complex health challenges [59, 60]. Our team encompassed researchers with a rich background, each contributing their unique expertise to different facets of the process. Neuropathology experts offered their understanding of tissue characteristics and pathological features to ensure the accuracy of slide annotations, relevance of quantitative measurements, and precise case classification, ultimately setting the ground truth for guidance of the algorithm training [61]. Clinicians provided clinical context for a detailed cohort characterization and real-world implications. Machine learning engineers designed and implemented the CNN model driving our automated quantification approach, harnessing the power of ML for accurate and efficient analyses, meeting healthcare system demands. Lastly, statisticians were instrumental in designing robust analytical strategies, ensuring the statistical validity of our findings. This collective synergy not only bolstered the technical rigor, but also fostered a holistic perspective that integrates computational innovations with clinicopathological insights [2]. Such collaboration advances research at the intersection of medical science and technology, yielding comprehensive insights bridging the gap between traditional diagnostic frameworks and modern computational techniques, thereby deepening phenotyping of AD.

Although these findings can significantly contribute to the field of dementia, several caveats merit mention as they contextualize the scope and applicability of our methodology. First, the retrospective nature of our study can be seen as a limitation: historical data were collected retrospectively from a single ADRC, and in some instances, information can be missing or incorrectly recorded, which can potentially create bias and inaccuracy. Assessments were conducted by multiple persons over the study’s timeframe, and were based on semi-quantitative methods, which can also have rater variability and might have impacted the classification of cases into ADNC groups. Additionally, the presence of high-score outliers in the dataset can impact the optimal functionality of the algorithm, particularly during the segmentation step. Furthermore, our analysis did not include a robust assessment of the algorithm’s response to tissue artifacts, such as tears, folds, or dust on the slide, which can potentially interfere with the final pathology quantification. Another limitation relates to the uniformity of our dataset; all items were scanned using a singular scanner and brain region, which may constrain the generalizability of our findings to different pre-analytic variables [62]. Additionally, the runtime for our algorithm of approximately 6 h per WSI using specialized hardware (GPUs), particularly in relation to the segmentation of GM and WM, may be comparatively slow when compared with other efforts. While our study provides detailed insights into select classifications of Aβ pathologies in the temporal lobe, care should be applied on generalizing results to other brain regions and can diminish the diversity of CAA (such as capillary, leptomeningeal) and plaques types (such as neuritic, compact). Future studies expanding the scope to include other brain regions and capturing the additional diversity of Aβ deposits, are necessary to enhance the generalizability and applicability of our machine learning-based quantification approach for deeper phenotyping.

Inherent to this study are key strengths underpinning the robustness and clinical relevance of our findings. This study performed a comprehensive detailed workflow leveraging ML techniques, advanced imaging analysis, and neuropathological expertise, providing a quantitative approach to evaluate AD pathology. A recent paper, focused on harmonizing newly generated digital measures with historical measures across multiple large autopsy-based studies had similar findings [63]. To the authors’ knowledge, this is the first ML-based study of Aβ-deposits in correlation with existing semi-quantitative established diagnostic criteria and the NIA-AA criteria in addition to clinical and genetic variables, demonstrating additional relevance and validation of the ML pipeline. The integration of traditional neuropathological expertise with modern computational techniques bridges the gap between established diagnostic practices and innovative technologies.

Our study confirms the validity of previously published ML pipelines for neuropathology analysis [24, 25]. These findings highlight the adaptability of the pipeline with minimal adjustments in a new, well-characterized cohort, yielding results of substantial clinicopathological reliability. We demonstrate the algorithm’s efficacy in stratifying cases and accurately classifying Aβ deposits, exhibiting strong concordance with existing pathological staging criteria and previous clinical assessments. Of significant importance is the crucial role postmortem brain evaluation and autopsy-based studies holds in clarifying disease mechanisms, acting as the foundational truth to steer translational research for therapeutic and precision medicine purposes. Moreover, our exploration opens the door to the prospect of stronger clinicopathological correlations, amalgamating the quantitative outcomes with imaging and biomarker data. Additionally, we hope this investigation will act as a catalyst, inspiring other research groups to forge collaborative partnerships that transcend institutional and disciplinary boundaries. By fostering multi-center and multidisciplinary collaborations, these partnerships can facilitate the validation of ML models in neuropathology, particularly across larger and more diverse datasets, ultimately augmenting the diagnostic ability of experts. This collective effort stands to advance the deepening in phenotyping of AD and accelerate progress in understanding its complex mechanisms.

### Electronic supplementary material

Below is the link to the electronic supplementary material.

Supplementary Material 1

Supplementary Material 2

## Data Availability

All codes related to plaque detection can be found in the GitHub repository (https://github.com/keiserlab/plaquebox-paper). All WM/GM segmentation codes, and the combined detection/segmentation pipeline codes are available in this GitHub repository (https://github.com/ucdrubinet/BrainSec*).* All listed GitHub repositories contain the full end-to-end pipeline, no outside code is needed to reproduce this study’s results.

## Abbreviations

AD: Alzheimer disease
ADRD: Alzheimer disease and related dementia
CAA: Cerebral amyloid angiopathy
CNN: Convolutional neural networks
GM: Gray matter
ML: Machine learning
WM: White matter
WSI: Whole slide imaging

## Data availability

The available data are located in Zenodo under DOI (10.5281/zenodo.10668642). All WSIs used in this study are available in their raw, de-identified form. Preprocessing, training and evaluation can be carried out using the codes listed in this manuscript.
