# Supplementary material for: Machine learning quantification of Amyloid-β deposits in the temporal lobe of 131 brain bank cases
Source: Acta Neuropathol Commun. 2024 Aug 17;12:134. doi: 10.1186/s40478-024-01827-7 (PMC11330038; doi:10.1186/s40478-024-01827-7)
Supplement: Supplementary file 1 — Supplementary Material 1 [file 40478_2024_1827_MOESM1_ESM.docx]

**Fig. S1** The boxplots depict the median A deposits in the white matter (WM), stratified by their assignments according to staging/scales (Thal Phase = Thal Amyloid Phase, Braak Stage = Braak Neurofibrillary Tangle Stage and CERAD score = CERAD Neuritic Plaque score). The horizontal line marks the median, the box encapsulates the interquartile range (IQR), and the whiskers extend to the smallest and largest observation within 1.5 times the IQR of the bottom and top of the box (* = p < 0.05; ** = p < 0.01; and *** = p < 0.001)


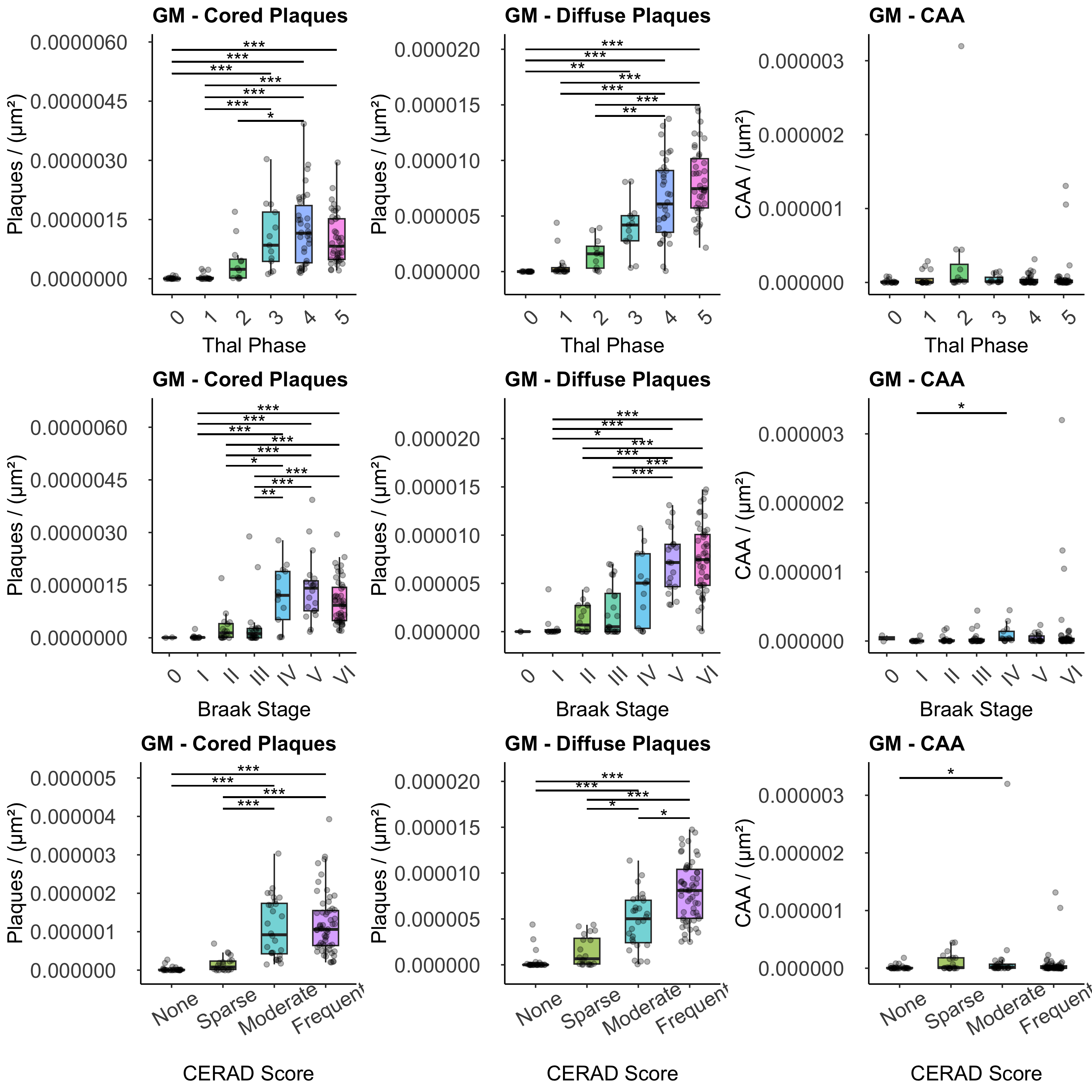


**Fig. S2** Boxplots depict the median A plaques (cored and diffuse) in the Gray Matter (GM), stratified by their assignments according to pathology groups. (Controls: Plaque Count = 0 and NIA-AA “ABC Score” = 0; All AD = ADNC Intermediate or High, mixed pathology present, Pure AD = ADNC Intermediate or High, with no TDP-43 or Lewy body disease (LBD) deposits). The horizontal line marks the median, the box encapsulates the interquartile range (IQR), and the whiskers extend to the smallest and largest observation within 1.5 times the IQR of the bottom and top of the box (* = p < 0.05; ** = p < 0.01; and *** = p < 0.001)

**Fig. S3** Boxplots depict the median A plaques (cored and diffuse) in the Gray Matter (GM), stratified by their assignments according to pathology group. (Pure = ADNC Intermediate or High, with no TDP-43 or LBD deposits, AD and TDP = ADNC Intermediate or High and presence of TDP-43 deposits; AD and LBD = ADNC Intermediate or High and presence of Lewy body disease (LBD) deposits). The horizontal line marks the median, the box encapsulates the interquartile range (IQR), and the whiskers extend to the smallest and largest observation within 1.5 times the IQR of the bottom and top of the box. AD and LBD and AD and TDP 43 groups are not mutually exclusive. There were no significant differences across groups (* = p < 0.05; ** = p < 0.01; and *** = p < 0.001)

**Fig. S4** Boxplots depict the median A plaques (cored and diffuse) in the Gray Matter (GM), stratified by their assignments according to pathology group. cases with and without the presence of Lewy body disease deposits (LBD group and no LBD group) and cases with and without the presence of TDP-43 inclusions (TDP-43 group and no TDP-43 group), regardless of Alzheimer Disease Neuropathologic Change (ADNC). The horizontal line marks the median, the box encapsulates the interquartile range (IQR), and the whiskers extend to the smallest and largest observation within 1.5 times the IQR of the bottom and top of the box. AD and LBD and AD and TDP 43 groups are not mutually exclusive (* = p < 0.05)
